# Supplementary material for: Limited window for donation of convalescent plasma with high live-virus neutralizing antibody titers for COVID-19 immunotherapy
Source: Commun Biol. 2021 Feb 24;4:267. doi: 10.1038/s42003-021-01813-y (PMC7904946; doi:10.1038/s42003-021-01813-y)
Supplement: Supplementary file 2 — Supplementary Information [file 42003_2021_1813_MOESM2_ESM.pdf]

Supplement materials:

Supplement Figures:

Fig. S1.

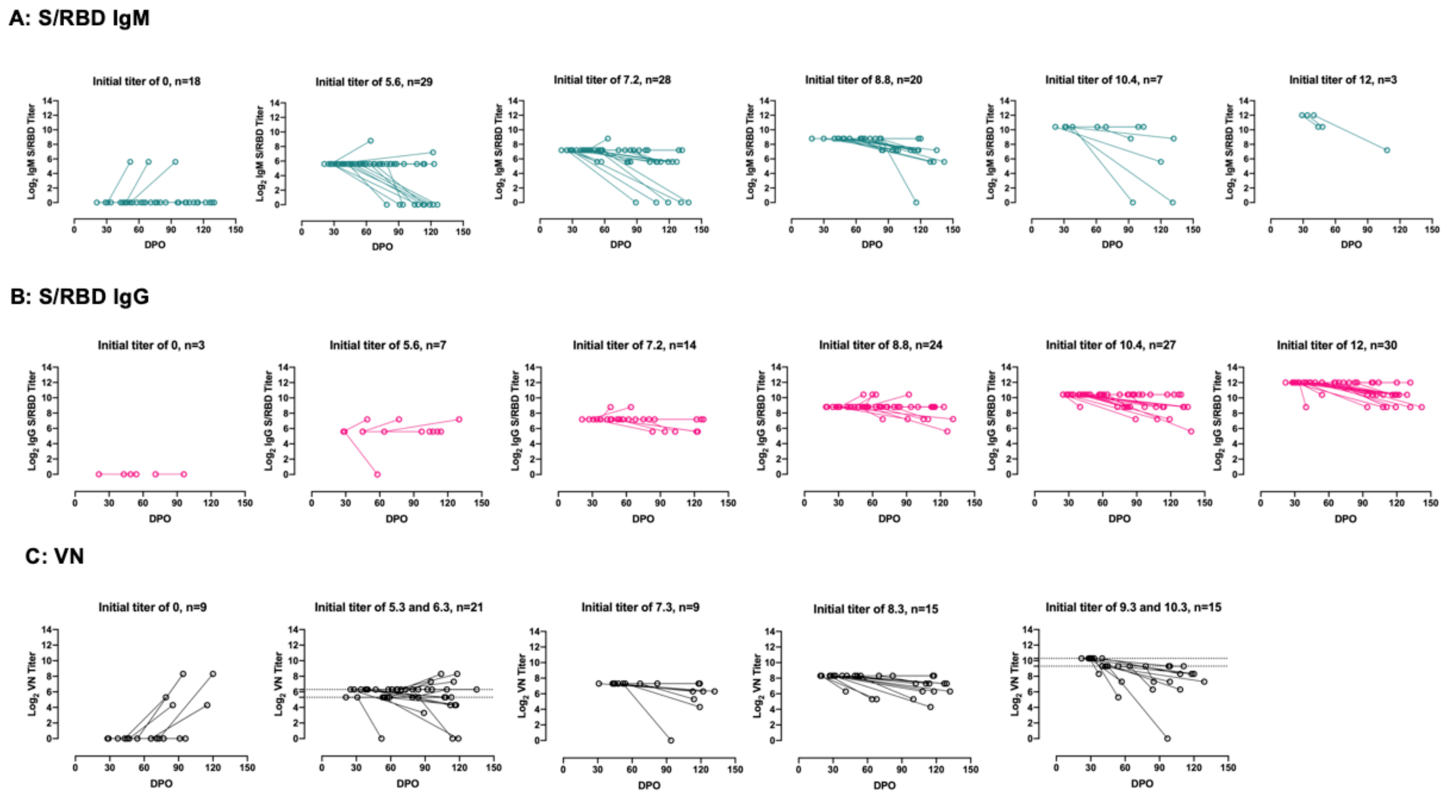

Fig. S1. Trajectories of (first and last donation only) (A) IgM, (B) IgG, and (C) VN antibody titers in subjects who donated plasma more than once. These data represent initial (Log_2) S/RBD IgM and IgG titers ≥5.3 remain stable or vary by one or two dilutions below or above the initial titer. A majority of individuals (33 out of 39) with initial (Log_2) VN titers ≥7.3 begin to drop beyond ~60 DPO.

Fig. S2.

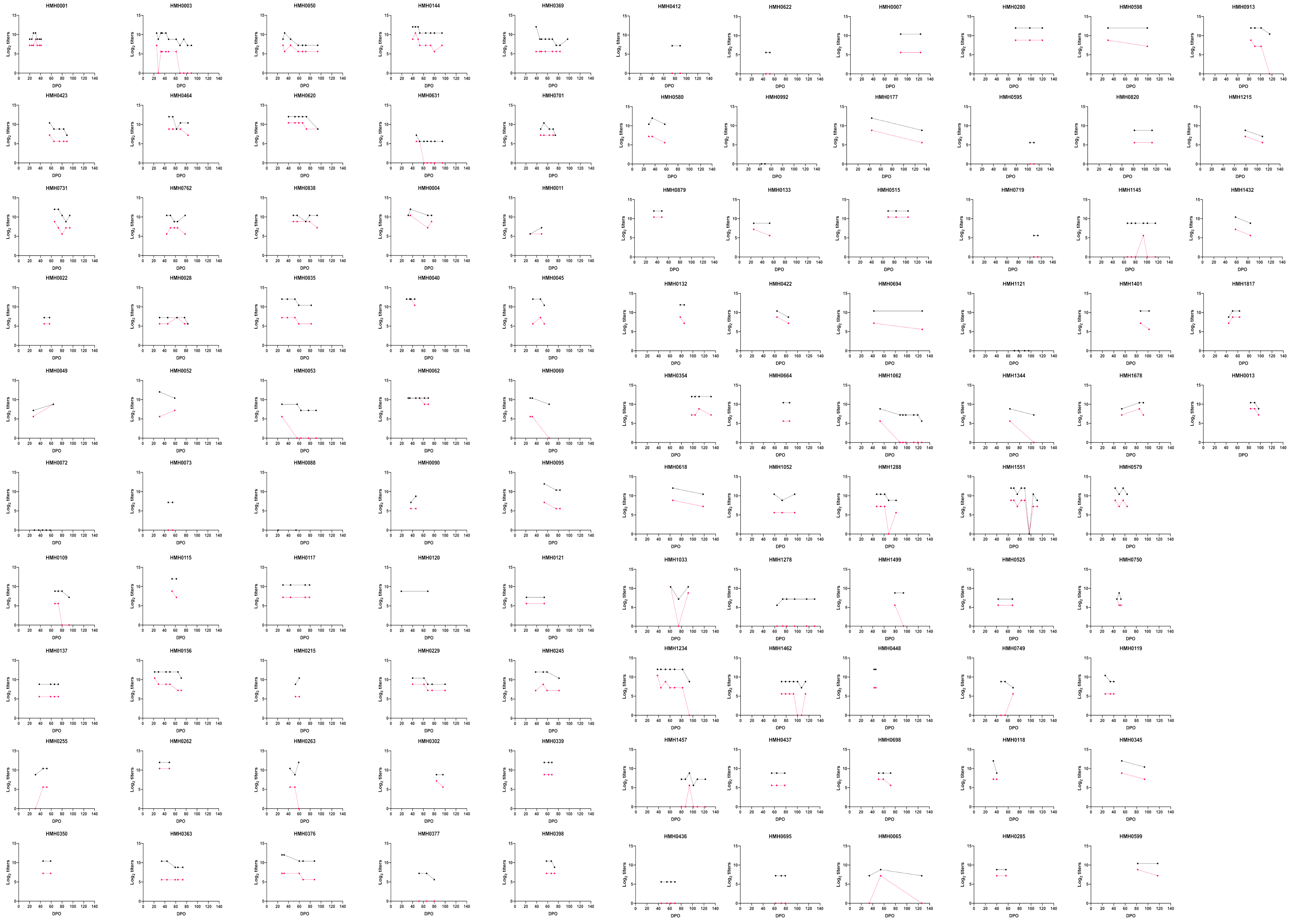

Fig. S2. Trajectories of IgM (pink) and IgG (black) antibody titers in subjects who donated plasma more than once.

Fig. S3.

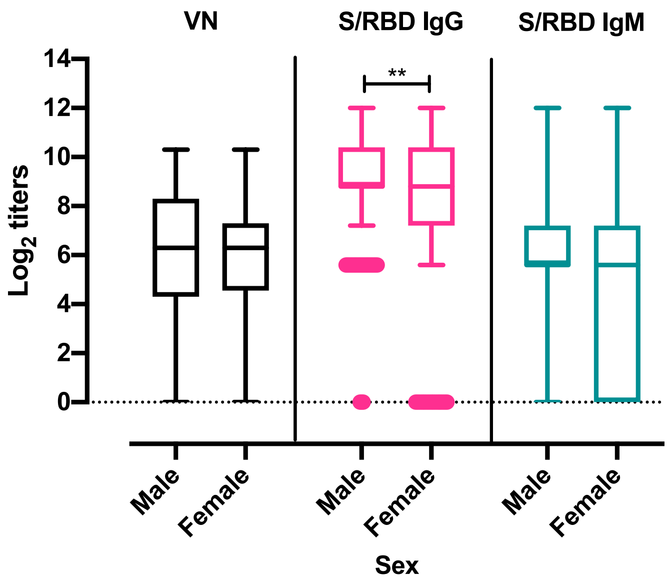

Fig. S3. Distribution of antibody titers against SARS-CoV-2 based on sex. Significant differences were observed between the S/RBD IgG titers of males (*n*= 327) and females (*n*= 213). (***P*<0.01, Unpaired t-test, two tailed). There were no differences observed in the IgM and VN titers of the study population when stratified by sex.

Fig. S4.

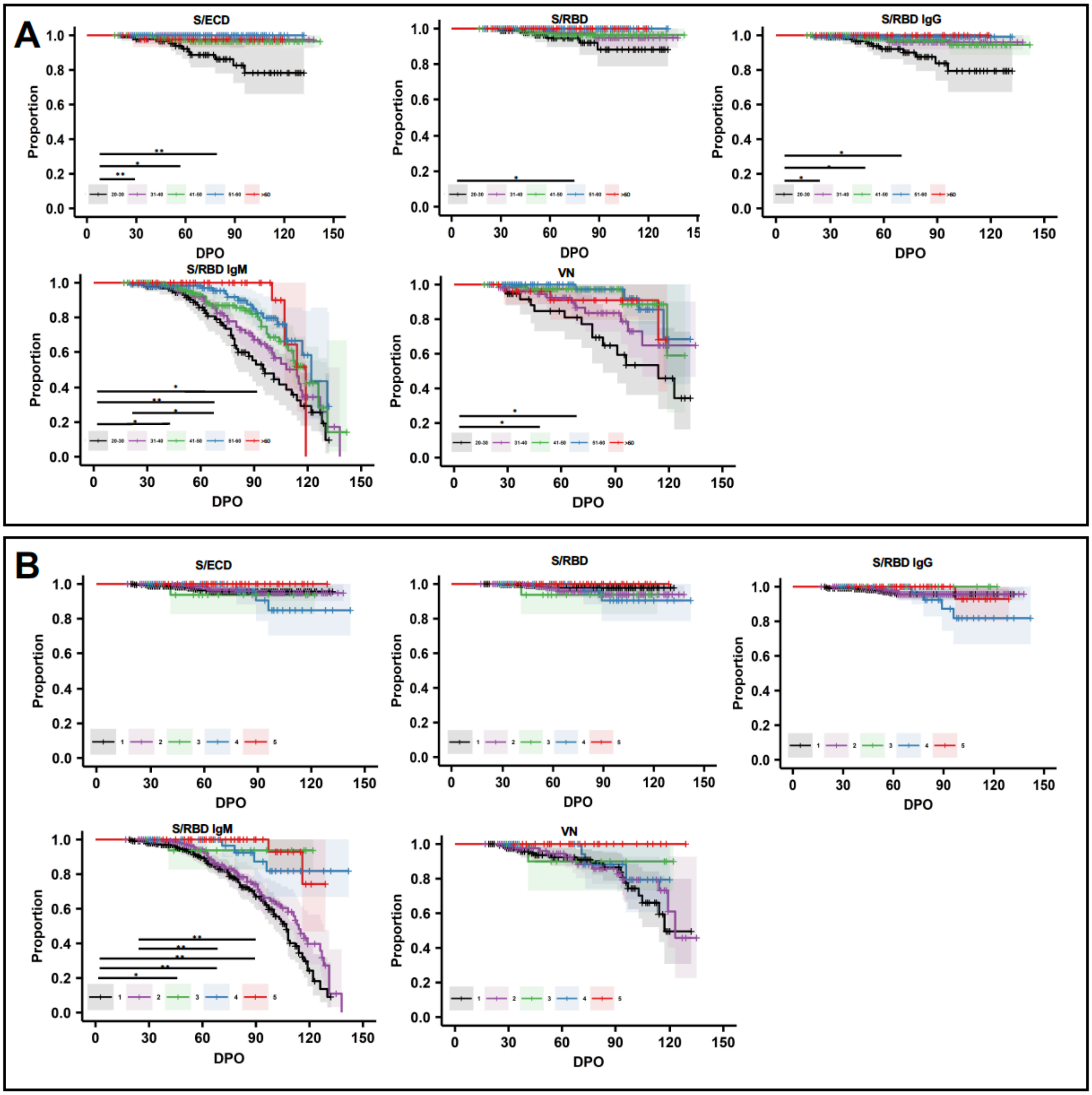

Fig. S4. Survival analysis of IgG, IgM and VN antibody titers to SARS-CoV-2 spike-receptor binding domain (S/RBD). These data represent survival analysis of SARS-CoV-2 spike ectodomain (S/ECD), SARS-CoV-2 spike-receptor binding domain (S/RBD), S/RBD IgM, S/RBD IgG, and neutralizing (VN) antibody titers in 175 convalescent individuals during the first 140 days post onset (DPO) of symptoms stratified by (A) age and (B) severity (Log-rank test, **P*<0.05, ***P*<0.01). Significant differences were observed in the titers of ELISAs between the age groups: 20-30 versus 31-40 (S/ECD ***P*<0.01, S/RBD IgG **P*<0.05); 20-30 versus 41-50 (S/ECD **P*<0.05, S/RBD IgG **P*<0.05, S/RBD IgM **P*<0.05, VN **P*<0.05); 20-30 versus 51-60 (S/ECD ***P*<0.01, S/RBD **P*<0.05, S/RBD IgG **P*<0.05, S/RBD IgM ***P*<0.01, VN **P*<0.05); 20-30 versus >60 (S/RBD IgM **P*<0.05); and 31-40 versus 51-60 (S/RBD IgM **P*<0.05). Significant differences were observed in the S/RBD IgM titers of the donors with the severity scores 1 versus 3 (**P*<0.05); 1 versus 4,5 (***P*<0.01); and 2 versus 4,5 (***P*<0.01).

Fig S5.

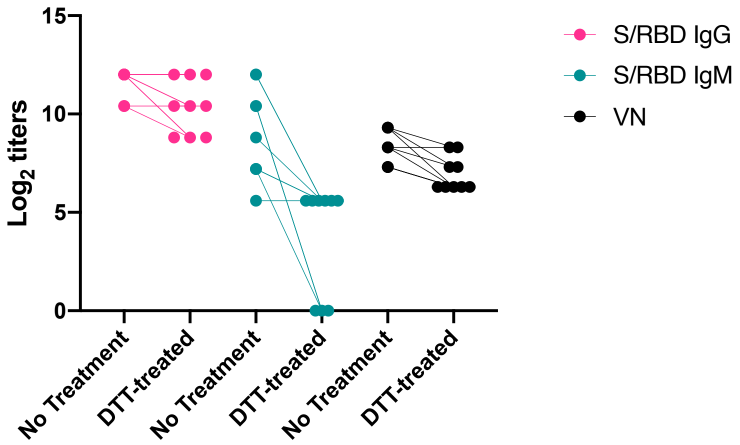

Fig S5. Antibody titers before and after 1,4-Dithiothreitol (DTT) treatment of convalescent plasma. Isotype specific antibody titers were quantified to determine the contribution of each antibody isotype- IgG and IgM in the virus neutralizing responses.

Fig. S6.

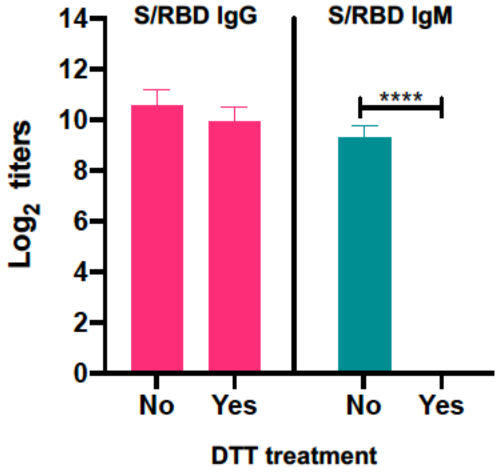

Fig. S6. Class specificity test for SARS-CoV-2 spike-receptor binding domain (S/RBD) isotype specific indirect ELISAs. 1,4-Dithiothreitol (DTT) treatment of convalescent plasma abrogates S/RBD IgM antibody titers but not IgG titers (n=10) (paired t test, *****P*<0.0001).

Fig. S7.

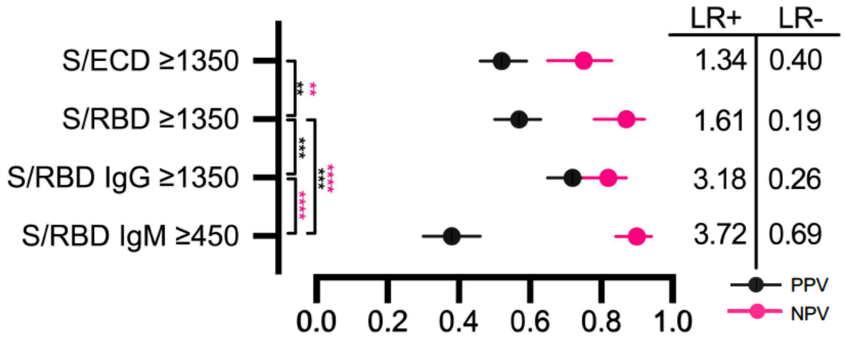

Fig. S7. Forest plot depicting the positive and negative predictive values for detection of SARS-CoV-2 spike-receptor binding domain (S/RBD), SARS-CoV-2 spike ectodomain (S/ECD), and S/RBD IgG titers ≥1350 using virus neutralization (VN) titer ≥160 as the standard. Likelihood ratios (LR) for each assay are shown on the right panel. P values were generated using the generalized score statistic for pairwise comparisons. For positive predictive values (PPV) S/ECD ≥1350 versus S/RBD ≥1350 ***P*<0.01; S/RBD ≥1350 versus S/RBD IgG ≥1350 ****P*<0.001; S/RBD IgG ≥1350 versus S/RBD IgM ≥450 P>0.05; S/RBD IgM ≥450 versus S/RBD ≥1350 ****P*<0.001. For negative predictive values (NPV) S/ECD ≥1350 versus S/RBD ≥1350 ***P*<0.01; S/RBD ≥1350 versus S/RBD IgG ≥1350 P>0.05; S/RBD IgG ≥1350 versus S/RBD IgM ≥450 *****P*<0.0001; S/RBD IgM ≥450 versus S/RBD 1350 *****P*<0.0001.

Supplement Tables:

Supplement Table 1: Convalescent plasma donor demographics and sample characteristics.

Subject	Age	Sex	Hospitalization	Severity	Dyspnea	DPO	VN titer	S/ECD titer	S/RBD titer	S/RBD IgG titer	S/RBD IgM titer
**0001**	44	M	NO	1	NO	20	320	150	1350	450	150
**0001**						24	640	-	-	450	150
**0001**						27	320	150	450	1350	150
**0001**						31	320	150	3200	1350	450
**0001**						34	320	150	3200	450	150
**0001**						38	160	450	450	450	150
**0001**						41	80	450	450	450	150
**0002**	54	M	NO	1	NO	28	40	50	150	150	50
**0003**	36	M	NO	1	NO	25	80	450	1350	1350	150
**0003**						28	80	150	450	450	0
**0003**						33	80	450	3200	1350	50
**0003**						35	0	450	450	1350	50
**0003**						42	20	150	450	1350	50
**0003**						47	10	1350	1350	450	50
**0003**						61	20	1350	1350	450	50
**0003**						68	0	450	1350	150	0
**0003**						75	40	1350	450	450	0
**0003**						82	20	1350	450	150	0
**0003**						89	10	1350	1350	150	0
**0004**	54	F	NO	2	YES	32	320	1350	1350	1350	1350
**0004**						36	640	1350	1350	4050	1350
**0004**						68	0	1350	1350	1350	150
**0004**						75	80	1350	1350	1350	450
**0004**						103	160	1350	1350	1350	150
**0004**						118	80	1350	1350	1350	150
**0004**						131	-	1350	1350	450	0
**0005**	58	M	NO	2	YES	91	-	150	150	150	0
**0007**	36	M	NO	1	NO	88	-	1350	1350	1350	50
**0007**						123	-	1350	1350	1350	50
**0009**	38	F	NO	2	YES	30	80	450	450	450	50
**0011**	67	F	NO	1	NO	28	0	0	50	50	50
**0011**						49	-	450	150	150	50
**0012**	46	F	NO	1	NO	30	320	150	450	150	150
**0013**	43	F	NO	1	NO	28	320	1350	3200	4050	450
**0016**	47	F	NO	1	NO	32	640	4050	4050	4050	1350
**0020**	41	F	NO	2	YES	17	20	50	200	50	50
**0022**	22	M	NO	1	NO	47	-	450	150	150	50
**0022**						57	-	450	1350	150	50
**0028**	23	M	NO	1	NO	31	20	150	150	150	50

Subject	Age	Sex	Hospitalization	Severity	Dyspnea	DPO	VN titer	S/ECD titer	S/RBD titer	S/RBD IgG titer	S/RBD IgM titer
**0028**						46	-	450	150	150	50
**0028**						63	-	0	450	150	150
**0028**						77	-	150	150	150	50
**0028**						83	0	450	450	50	50
**0029**	66	F	NO	1	NO	22	80	150	450	4050	150
**0032**	65	M	NO	2	YES	25	320	450	4050	4050	1350
**0035**	50	M	NO	2	YES	28	320	1350	3200	4050	150
**0035**						38	640	1350	1350	4050	150
**0035**						52	-	1350	1350	4050	150
**0035**						59	160	1350	1350	1350	50
**0035**						82	640	1350	1350	1350	50
**0035**						108	80	1350	1350	450	0
**0040**	52	M	NO	2	YES	29	1280	-	-	4050	4050
**0040**						35	320	4050	4050	4050	4050
**0040**						37	320	1350	4050	4050	4050
**0040**						44	-	4050	4050	4050	1350
**0045**	23	F	NO	1	NO	33	1280	4050	1350	4050	50
**0045**						47	-	1350	1350	4050	150
**0045**						54	40	1350	1350	1350	50
**0049**	57	F	NO	1	NO	27	320	150	450	150	50
**0049**						64	40	1350	1350	450	450
**0050**	41	M	NO	2	YES	30	320	1350	1350	450	150
**0050**						33	0	150	150	1350	50
**0050**						44	20	1350	450	450	150
**0050**						58	20	1350	1350	150	50
**0050**						66	40	1350	450	150	50
**0050**						72	20	1350	1350	150	50
**0050**						94	20	1350	1350	150	50
**0050**						102	20	1350	1350	150	50
**0050**						108	-	1350	450	450	50
**0050**						115	20	1350	450	150	50
**0050**						131	-	1350	1350	150	0
**0051**	50	F	NO	1	NO	30	160	150	450	150	150
**0052**	27	F	YES	3	YES	31	160	1350	1350	4050	50
**0052**						59	-	1350	1350	1350	150
**0052**						87	160	1350	1350	1350	150
**0052**						122	80	1350	1350	1350	150
**0053**	29	M	NO	2	YES	28	0	450	450	450	50
**0053**	30	M	NO	2	YES	56	-	450	450	450	0
**0053**						63	-	450	450	150	0
**0053**						77	0	1350	1350	150	0
**0053**						91	0	150	50	150	0

Subject	Age	Sex	Hospitalization	Severity	Dyspnea	DPO	VN titer	S/ECD titer	S/RBD titer	S/RBD IgG titer	S/RBD IgM titer
**0055**	61	M	YES	3	YES	33	320	1350	3200	1350	150
**0057**	44	F	NO	2	YES	34	160	450	450	450	150
**0058**	36	M	NO	2	YES	92	-	1350	1350	1350	50
**0062**	24	F	NO	1	NO	32	320	1350	1350	1350	1350
**0062**						35	1280	1350	1350	1350	1350
**0062**						46	-	1350	1350	1350	1350
**0062**						53	-	1350	1350	1350	1350
**0062**						62	-	1350	1350	1350	450
**0062**						69	320	1350	1350	1350	450
**0062**						101	80	1350	1350	450	450
**0062**						118	160	1350	1350	450	450
**0062**						132	80	1350	1350	450	450
**0065**	50	M	NO	1	NO	34	-	1350	1350	150	0
**0065**						54	-	1350	1350	450	150
**0065**						126	-	450	450	150	0
**0069**	49	F	NO	1	NO	28	80	450	450	1350	50
**0069**						32	80	450	450	1350	50
**0069**						63	-	50	450	450	0
**0069**						108	40	1350	450	150	0
**0070**	37	F	NO	2	YES	38	160	450	1350	4050	50
**0072**	23	F	NO	1	NO	29	0	0	0	50	0
**0072**						37	0	50	50	0	0
**0072**						44	-	0	0	0	0
**0072**						51	-	0	0	0	0
**0072**						58	-	0	0	0	0
**0073**	39	F	NO	2	YES	47	0	150	150	150	0
**0073**						55	-	450	450	150	0
**0073**						85	20	1350	450	150	0
**0077**	59	F	NO	1	NO	71	-	1350	1350	1350	150
**0081**	64	F	NO	2	YES	52	-	450	1350	1350	150
**0088**	29	F	NO	1	NO	21	20	0	50	0	0
**0088**						54	-	0	50	0	0
**0089**	42	F	NO	2	YES	38	160	450	450	4050	150
**0090**	33	M	NO	1	NO	37	1280	150	150	150	50
**0090**						46	-	1350	1350	450	50
**0095**	61	F	NO	1	NO	54	160	1350	1350	4050	150
**0095**						76	-	1350	1350	1350	50
**0095**						83	-	1350	1350	1350	50
**0095**						119	20	1350	1350	450	0
**0096**	44	F	NO	1	NO	59	-	1350	1350	1350	50
**0099**	54	F	NO	1	NO	20	20	50	50	0	0

**0109**	33	F	NO	1	NO	67	160	1350	1350	450	50
**0109**						73		1350	1350	450	50
**0109**						80	160	1350	1350	450	0
**0109**						93	0	450	150	450	0
**Subject**	**Age**	**Sex**	**Hospitalization**	**Severity**	**Dyspnea**	**DPO**	**VN titer**	**S/ECD titer**	**S/RBD titer**	**S/RBD IgG titer**	**S/RBD IgM titer**
**0109**						100	-	450	450	450	0
**0109**						108	-	1350	450	450	0
**0109**						114	40	1350	450	450	0
**0112**	47	F	NO	1	NO	32	40	450	450	450	150
**0113**	52	F	NO	1	NO	29	40	1350	150	150	0
**0115**	70	M	YES	5	NO	54	320	1350	1350	4050	450
**0115**						62	-	1350	1350	4050	150
**0115**						102	640	1350	1350	1350	150
**0115**						118	320	1350	1350	1350	150
**0116**	27	M	NO	1	NO	32	20	450	450	450	50
**0117**	27	F	NO	2	YES	30	320	1350	1350	1350	150
**0117**						44	-	1350	1350	1350	150
**0117**						71	-	1350	1350	1350	150
**0117**						79	160	1350	1350	1350	150
**0117**						85	-	1350	1350	1350	150
**0117**						129	160	1350	1350	1350	150
**0118**	50	F	NO	1	NO	34	320	1350	450	4050	150
**0118**						40	-	1350	1350	450	150
**0119**	35	F	NO	1	NO	25	0	450	450	1350	50
**0119**						34	-	1350	1350	450	50
**0119**						40	-	1350	450	450	50
**0120**	41	F	NO	1	NO	19	320	450	3200	450	450
**0120**						68	40	1350	1350	450	450
**0121**	51	F	NO	1	NO	21	40	150	200	150	50
**0121**						54	40	450	450	150	50
**0132**	61	M	YES	5	YES	78	640	1350	1350	4050	450
**0132**						85	160	1350	1350	4050	150
**0133**	51	M	NO	2	YES	25	-	150	450	450	150
**0133**						53	-	1350	1350	450	50
**0135**	47	F	NO	2	YES	32	320	4050	4050	4050	150
**0137**	53	F	NO	1	NO	38	80	450	3200	450	50
**0137**						59	-	450	1350	450	50
**0137**						66	-	1350	1350	450	50
**0137**						73	80	1350	1350	450	50
**0140**	54	M	NO	1	NO	98	-	1350	1350	450	50
**0143**	49	F	NO	2	YES	37	160	1350	3200	4050	150
**0144**	48	M	YES	5	YES	40	640	1350	3200	4050	450
**0144**						45	640	4050	1350	4050	1350

Subject	Age	Sex	Hospitalization	Severity	Dyspnea	DPO	VN titer	S/ECD titer	S/RBD titer	S/RBD IgG titer	S/RBD IgM titer
**0144**						50	320	450	1350	4050	450
**0144**						53	1280	4050	4050	1350	150
**0144**						64	320	1350	1350	1350	150
**0144**						73	80	1350	1350	1350	150
**0144**						80	160	1350	1350	1350	50
**0144**						94	80	1350	1350	1350	150
**0144**						115	160	1350	1350	1350	50
**0144**						121	-	1350	450	1350	50
**0144**						129	160	1350	1350	1350	50
**0156**	59	M	NO	1	NO	22	1280	4050	1350	4050	1350
**0156**						29	1280	1350	1350	4050	450
**0156**						43	80	1350	1350	4050	450
**0156**						50	-	1350	1350	4050	450
**0156**						65	-	1350	1350	4050	150
**0156**						71	160	1350	1350	1350	150
**0156**						85	-	1350	1350	1350	50
**0156**						92	-	1350	1350	1350	50
**0156**						99	160	1350	1350	1350	50
**0156**						106	-	1350	1350	1350	50
**0156**						113	-	1350	1350	1350	50
**0156**						120	320	1350	1350	1350	50
**0158**	33	M	NO	2	YES	26	40	150	200	450	50
**0159**	23	F	NO	2	YES	46	-	4050	4050	4050	450
**0162**	51	F	YES	3	YES	34	160	150	450	450	50
**0177**	55	M	NO	1	NO	44	160	4050	4050	4050	450
**0177**						132	80	1350	1350	450	50
**0215**	38	M	NO	2	YES	53	-	1350	1350	450	50
**0215**						60	-	1350	1350	1350	50
**0229**	32	M	NO	2	YES	40	80	450	1350	1350	450
**0229**						61	320	1350	1350	1350	450
**0229**						68	-	1350	1350	450	150
**0229**						76	-	1350	1350	450	150
**0229**						100	160	1350	1350	450	150
**0229**						110	-	1350	1350	450	150
**0229**						117	160	1350	1350	450	50
**0229**						135	80	1350	1350	450	150
**0234**	40	M	NO	2	YES	27	40	150	150	150	150
**0245**	51	M	YES	5	YES	38	320	1350	4050	4050	150
**0245**						52	-	1350	1350	4050	450
**0245**						59	-	1350	1350	4050	150
**0245**						81	320	1350	1350	1350	150
**0245**						102	160	1350	1350	1350	50

**0249**	56	M	NO	1	NO	22	320	4050	4050	4050	150
**0255**	40	M	NO	2	YES	31	40	450	450	450	0
**0255**						45	-	1350	1350	1350	50
**0255**						52	0	1350	1350	1350	50
**Subject**	**Age**	**Sex**	**Hospitalization**	**Severity**	**Dyspnea**	**DPO**	**VN titer**	**S/ECD titer**	**S/RBD titer**	**S/RBD IgG titer**	**S/RBD IgM titer**
**0260**	44	M	NO	2	YES	24	1280	4050	1350	1350	1350
**0262**	36	F	YES	4	YES	31	1280	4050	4050	4050	1350
**0262**						49	-	1350	1350	4050	1350
**0262**						99	640	1350	1350	4050	1350
**0263**	20	M	NO	1	NO	43	0	1350	1350	1350	50
**0263**						52	-	150	1350	450	50
**0263**						59	-	1350	450	450	0
**0263**						79	40	1350	1350	450	0
**0265**	53	F	NO	2	YES	31	320	4050	4050	4050	150
**0280**	37	M	YES	4	YES	73	0	1350	1350	4050	450
**0280**						98	320	1350	1350	4050	450
**0280**						120	320	1350	1350	4050	450
**0284**	35	F	NO	4	YES	53	-	1350	1350	1350	150
**0285**	51	F	NO	1	NO	40	-	1350	1350	450	150
**0285**						56	-	1350	1350	450	150
**0287**	40	F	NO	2	YES	56	-	50	0	0	0
**0301**	59	M	NO	1	NO	83	-	1350	1350	1350	450
**0301**						90	-	1350	1350	1350	450
**0301**						97	-	1350	1350	450	150
**0302**	25	M	NO	1	NO	84	80	1350	1350	450	150
**0302**						96	80	450	150	450	50
**0302**						112	-	450	1350	450	50
**0313**	78	M	YES	3	YES	36	160	4050	4050	4050	50
**0339**	31	M	YES	3	YES	54	640	150	1350	4050	450
**0339**						62	-	1350	1350	4050	450
**0339**						68	160	1350	1350	4050	450
**0339**						82	-	1350	1350	1350	150
**0339**						89	-	1350	1350	1350	150
**0339**						96	160	1350	1350	1350	150
**0339**						110	-	1350	1350	1350	150
**0339**						118	320	1350	1350	1350	150
**0345**	62	M	NO	1	NO	54	0	1350	1350	4050	450
**0345**						94	320	1350	1350	1350	150
**0350**	53	M	NO	1	NO	45	160	1350	1350	1350	150
**0350**						59	-	1350	1350	1350	150
**0350**						82	160	1350	1350	450	50
**0354**	59	M	YES	4	YES	98	640	1350	1350	4050	150
**0354**						104	-	1350	1350	4050	150

**0354**						111	640	1350	1350	4050	450
**0354**						132	-	1350	1350	4050	150
**0363**	56	M	NO	1	NO	34	80	450	1350	1350	50
**0363**						44	-	1350	1350	1350	50
**Subject**	**Age**	**Sex**	**Hospitalization**	**Severity**	**Dyspnea**	**DPO**	**VN titer**	**S/ECD titer**	**S/RBD titer**	**S/RBD IgG titer**	**S/RBD IgM titer**
**0363**						59	-	1350	1350	450	50
**0363**						64	-	1350	1350	450	50
**0363**						73	80	1350	1350	450	50
**0363**						86	-	450	1350	450	0
**0363**						100	20	1350	1350	450	50
**0363**						107	40	1350	450	450	50
**0367**	58	F	NO	2	YES	89	-	1350	1350	4050	450
**0368**	37	F	NO	2	YES	29	160	450	1350	4050	50
**0369**	41	M	NO	1	NO	39	80	150	450	1350	50
**0369**						46	40	450	450	450	50
**0369**						49	40	1350	1350	450	50
**0369**						56	80	1350	1350	450	50
**0369**						63	20	1350	1350	450	0
**0369**						69	20	1350	450	450	50
**0369**						76	20	1350	450	150	50
**0369**						83	10	1350	1350	150	50
**0369**						97	20	450	450	450	0
**0369**						104	-	450	150	150	0
**0369**						112	20	1350	450	150	0
**0369**	41	M	NO	1	NO	119	-	450	450	150	0
**0376**	52	M	YES	4	YES	28	1280	1350	4050	4050	150
**0376**						32	160	4050	4050	4050	150
**0376**						60	-	1350	1350	1350	150
**0376**						67	80	1350	1350	1350	50
**0376**						88	160	1350	1350	1350	50
**0376**						108	80	1350	1350	450	50
**0377**	49	M	NO	2	YES	52	160	450	1350	150	0
**0377**						66	-	50	450	150	0
**0377**						80	-	450	450	50	0
**0377**						94	0	450	450	50	50
**0385**	45	M	NO	2	YES	63	-	0	0	0	0
**0398**	55	F	YES	4	YES	58	-	1350	1350	1350	150
**0398**						67	-	1350	1350	1350	150
**0398**						73	-	1350	1350	450	150
**0412**	53	M	NO	1	NO	75	20	1350	450	150	0
**0412**						89	-	1350	1350	150	0
**0412**						103	0	1350	450	50	0
**0419**	40	M	NO	2	YES	68	--	1350	1350	1350	150

Subject	Age	Sex	Hospitalization	Severity	Dyspnea	DPO	VN titer	S/ECD titer	S/RBD titer	S/RBD IgG titer	S/RBD IgM titer
**0422**	41	M	NO	1	NO	64	640	1350	1350	1350	450
**0422**						84	80	1350	450	450	150
**0423**	39	M	NO	2	YES	57	40	450	1350	1350	150
**0423**						65	20	1350	1350	450	50
**0423**						75	20	50	1350	450	50
**0423**						83	20	1350	1350	450	50
**0423**						89	20	450	1350	150	50
**0423**						117	20	450	450	150	0
**0423**						127	-	1350	450	150	0
**0423**						131	-	1350	1350	150	0
**0423**						138	-	450	1350	50	0
**0430**	44	M	YES	4	YES	35	1280	4050	4050	4050	150
**0436**	32	F	NO	1	NO	45	640	1350	1350	50	0
**0436**						55	-	1350	1350	50	0
**0436**						62	-	450	1350	50	0
**0436**						69	-	1350	1350	50	0
**0436**						90	-	1350	1350	50	0
**0436**						97	0	1350	1350	50	0
**0437**	49	M	NO	1	NO	55	40	1350	1350	450	50
**0437**						64	-	1350	1350	450	50
**0437**						78	-	1350	1350	450	50
**0437**						113	40	1350	1350	450	50
**0448**	49	M	NO	2	YES	43	160	1350	1350	4050	150
**0448**						46	-	1350	1350	4050	150
**0448**						91	160	1350	1350	1350	150
**0448**						105	-	1350	1350	4050	150
**0448**						112	-	1350	1350	1350	50
**0448**						119	160	1350	1350	1350	50
**0462**	47	F	NO	2	YES	61	-	450	1350	450	0
**0464**	31	F	NO	2	YES	48	160	1350	1350	4050	450
**0464**						55	160	1350	1350	4050	450
**0464**						62	80	1350	1350	450	450
**0464**						69	80	1350	1350	1350	450
**0464**						83	80	1350	1350	1350	150
**0464**						90	-	1350	1350	1350	450
**0464**						118	160	1350	1350	1350	450
**0479**	56	F	NO	1	NO	79	-	1350	1350	4050	450
**0488**	37	F	NO	1	NO	62	-	150	150	0	0
**0515**	58	M	YES	4	YES	69	80	1350	1350	4050	1350
**0515**						83	-	1350	1350	4050	1350
**0515**						104	320	1350	1350	4050	1350
**0524**	35	F	YES	3	YES	44	-	1350	1350	4050	450

**0525**	33	F	NO	2	YES	43	-	450	50	150	50
**0525**						68	-	50	450	150	50
**0526**	74	M	NO	2	YES	45	-	1350	1350	1350	150
**0530**	39	F	NO	1	NO	71	-	1350	1350	450	50
**Subject**	**Age**	**Sex**	**Hospitalization**	**Severity**	**Dyspnea**	**DPO**	**VN titer**	**S/ECD titer**	**S/RBD titer**	**S/RBD IgG titer**	**S/RBD IgM titer**
**0533**	32	F	NO	1	NO	47	-	450	450	150	0
**0548**	40	M	NO	4	NO	52	-	1350	1350	4050	150
**0554**	68	F	NO	1	NO	57	-	1350	1350	4050	1350
**0576**	50	F	YES	3	YES	41	0	0	0	50	0
**0579**	70	M	NO	2	YES	43	640	1350	1350	4050	450
**0579**						50	-	1350	1350	1350	150
**0579**						57	-	1350	1350	4050	450
**0579**						64	80	1350	1350	1350	150
**0579**						99	160	1350	1350	1350	150
**0580**	43	F	YES	3	YES	29	1280	4050	4050	1350	150
**0580**						35	1280	4050	4050	4050	150
**0580**						57	160	1350	1350	1350	50
**0581**	50	M	YES	4	YES	108	-	1350	1350	150	50
**0591**	51	F	NO	1	NO	44	-	450	1350	450	150
**0595**	26	F	NO	2	YES	104	-	150	150	50	0
**0595**						111	-	150	150	50	0
**0598**	46	M	YES	4	YES	30	1280	4050	4050	4050	450
**0598**						99	640	1350	1350	4050	150
**0599**	42	M	YES	3	YES	82	320	1350	1350	1350	450
**0599**						117	320	1350	1350	1350	150
**0605**	54	F	NO	1	NO	58	-	1350	1350	1350	150
**0610**	48	F	NO	2	YES	64	-	1350	1350	150	0
**0612**	36	F	NO	1	NO	69	-	1350	1350	50	0
**0618**	58	F	YES	5	YES	65	80	1350	1350	4050	450
**0618**						118	320	1350	1350	1350	150
**0620**	59	M	YES	5	YES	40	1280	4050	4050	4050	4050
**0620**						52	320	1350	1350	4050	1350
**0620**						59	320	1350	1350	4050	1350
**0620**						66	160	1350	1350	4050	1350
**0620**						73	160	1350	1350	4050	450
**0620**						94	320	1350	1350	450	450
**0620**						101	--	1350	1350	4050	150
**0620**						108	320	1350	1350	1350	150
**0622**	20	F	NO	1	NO	45	0	450	150	50	0
**0622**						52	-	450	150	50	0
**0622**						77	0	1350	450	150	0
**0631**	58	M	NO	1	NO	47	20	150	450	150	50

**0631**						53	20	150	1350	50	50
**0631**						61	20	1350	1350	50	0
**0631**						67	20	1350	150	50	0
**0631**						74	10	450	1350	50	0
**0631**						81	20	450	450	50	0
**Subject**	**Age**	**Sex**	**Hospitalization**	**Severity**	**Dyspnea**	**DPO**	**VN titer**	**S/ECD titer**	**S/RBD titer**	**S/RBD IgG titer**	**S/RBD IgM titer**
**0631**						95	0	450	450	50	0
**0631**						108	-	150	50	50	0
**0631**						117	0	450	450	50	0
**0631**						122	-	450	150	50	0
**0633**	63	M	YES	4	YES	79	-	1350	1350	1350	150
**0634**	53	M	YES	5	YES	33	1280	4050	4050	4050	450
**0636**	30	M	NO	2	YES	64	-	1350	1350	1350	450
**0664**	46	M	NO	1	NO	75	-	1350	1350	1350	50
**0664**						86	-	1350	1350	1350	50
**0694**	30	M	NO	2	YES	42	320	1350	1350	1350	150
**0694**						127	160	1350	1350	1350	50
**0695**	22	F	NO	2	YES	62	0	0	1350	150	0
**0695**						72	-	450	1350	150	0
**0695**						79	-	450	450	150	0
**0695**						114	0	450	150	150	0
**0695**						128	-	450	450	150	0
**0698**	32	M	NO	2	YES	51	320	1350	1350	450	150
**0698**						59	-	1350	1350	450	150
**0698**						72	-	1350	450	450	50
**0698**						100	40	1350	150	450	150
**0699**	61	M	YES	3	NO	35	640	4050	450	450	450
**0701**	41	M	NO	1	NO	47	80	1350	1350	450	150
**0701**						53	40	1350	1350	1350	150
**0701**						63	40	1350	1350	450	150
**0701**						70	40	1350	1350	450	150
**0701**						74	80	1350	1350	150	150
**0701**						105	20	1350	450	450	50
**0701**						109	-	1350	1350	50	0
**0701**						116	20	1350	450	450	50
**0701**						123	-	1350	1350	450	50
**0719**	63	M	NO	1	NO	107	-	1350	1350	50	0
**0719**						114	-	1350	1350	50	0
**0720**	39	M	NO	2	YES	63	-	50	0	0	0
**0731**	50	M	YES	4	YES	66	80	1350	1350	4050	450
**0731**						73	40	1350	1350	4050	150
**0731**						80	40	1350	1350	1350	50
**0731**						87	80	1350	1350	450	150

**0731**						94	40	1350	1350	1350	150
**0731**						101	-	1350	1350	4050	150
**0731**						115	160	1350	1350	1350	150
**0731**						128	-	1350	1350	1350	150
**0731**						142	-	1350	1350	450	50
**Subject**	**Age**	**Sex**	**Hospitalization**	**Severity**	**Dyspnea**	**DPO**	**VN titer**	**S/ECD titer**	**S/RBD titer**	**S/RBD IgG titer**	**S/RBD IgM titer**
**0749**	45	M	NO	1	NO	48	-	450	1350	450	0
**0749**						55	-	450	1350	450	0
**0749**						69	-	450	450	150	50
**0750**	29	M	NO	2	YES	46	-	450	150	150	150
**0750**						50	-	450	150	450	50
**0750**						53	-	150	1350	150	50
**0750**						123	0	450	450	50	50
**0759**	48	F	NO	2	YES	45	-	1350	1350	450	150
**0762**	47	M	NO	2	YES	44	160	450	1350	1350	50
**0762**						51	40	1350	1350	1350	150
**0762**						58	80	1350	1350	450	150
**0762**						64	160	1350	1350	450	150
**0762**						78	160	1350	1350	1350	50
**0762**						85	-	1350	1350	150	0
**0762**						92	40	1350	1350	450	0
**0762**						113	80	1350	1350	450	0
**0786**	46	F	NO	2	YES	48	-	450	1350	1350	150
**0789**	55	F	NO	2	YES	62	-	1350	1350	1350	150
**0796**	45	M	YES	5	YES	52	-	1350	1350	1350	450
**0820**	56	F	NO	1	NO	82	-	1350	1350	450	50
**0820**						113	-	1350	1350	450	50
**0834**	48	M	NO	2	YES	60	-	1350	1350	4050	450
**0835**	52	F	YES	5	YES	61	-	1350	1350	4050	4050
**0838**	74	F	NO	1	NO	49	320	1350	1350	1350	450
**0838**							160	1350	1350	1350	450
**0838**							160	1350	1350	450	450
**0838**							80	1350	1350	1350	450
**0838**							320	1350	1350	1350	150
**0838**							-	1350	1350	1350	150
**0838**							160	1350	1350	450	150
**0850**	34	F	NO	2	YES	67	-	1350	1350	1350	150
**0879**	50	M	YES	4	YES	34	160	4050	4050	4050	4050
**0879**							-	1350	1350	4050	1350
**0905**	41	F	NO	1	NO	57	-	1350	1350	150	0
**0913**	34	F	YES	5	YES	83	-	1350	1350	4050	450
**0913**						90	-	1350	1350	4050	150
**0913**						101	-	1350	1350	4050	150

**0913**						116	-	1350	1350	1350	0
**0933**	67	M	NO	1	NO	65	-	1350	1350	4050	450
**0970**	29	F	NO	2	YES	56	-	1350	1350	1350	50
**0992**	45	F	NO	2	YES	43	-	0	0	0	0

Subject	Age	Sex	Hospitalization	Severity	Dyspnea	DPO	VN titer	S/ECD titer	S/RBD titer	S/RBD IgG titer	S/RBD IgM titer
**0992**						49	-	0	0	0	0
**1033**	33	F	NO	1	NO	61	80	1350	1350	1350	1350
**1033**						75	-	1350	450	150	0
**1033**						92	80	1350	1350	1350	450
**1052**	28	F	NO	2	YES	59	40	1350	1350	1350	50
**1052**						73	-	150	1350	450	50
**1052**						95	160	1350	1350	1350	50
**1062**	44	F	NO	2	YES	53	40	1350	1350	450	50
**1062**						88	-	1350	1350	150	0
**1062**						93	20	450	150	150	0
**1062**						98	-	450	450	150	0
**1062**						112	-	450	450	150	0
**1062**						119	0	1350	150	150	0
**1062**						126	-	1350	1350	50	0
**1121**	28	F	YES	4	YES	71	0	1350	1350	0	0
**1121**						78	-	0	0	0	0
**1121**						89	-	0	0	0	0
**1121**						96	0	0	50	0	0
**1145**	39	F	NO	2	YES	66	0	1350	1350	450	0
**1145**						73	-	1350	1350	450	0
**1145**						80	-	1350	450	450	0
**1145**						94	40	1350	1350	450	50
**1145**						101	-	1350	1350	450	0
**1145**						115	20	1350	1350	450	0
**1215**	54	M	NO	1	NO	79	40	1350	1350	450	150
**1215**						109	80	1350	1350	150	50
**1234**	49	F	NO	2	YES	38	-		1350	4050	1350
**1234**						44	-	1350	1350	4050	150
**1234**						52	-	1350	1350	4050	450
**1234**						60	-	1350	1350	4050	150
**1234**						68	-	1350	1350	4050	150
**1234**						82	-	1350	1350	4050	150
**1234**						94	-	1350	1350	450	0
**1278**	23	M	NO	1	NO	64	-	50	1350	50	0
**1278**						74	-	1350	1350	150	0
**1278**						81	-	1350	1350	150	0
**1278**						95	-	1350	450	150	0
**1278**						116	-	1350	450	150	0

Subject	Age	Sex	Hospitalization	Severity	Dyspnea	DPO	VN titer	S/ECD titer	S/RBD titer	S/RBD IgG titer	S/RBD IgM titer
**1278**						130	-	1350	450	150	0
**1288**	27	F	NO	2	YES	47	-	1350	1350	1350	150
**1288**						54	-	1350	1350	1350	150
**1288**						61	-	1350	1350	1350	150
**1288**						68	-	1350	450	450	0
**1288**						81	80	1350	1350	450	50
**1344**	32	F	NO	1	NO	63	20	1350	450	450	50
**1344**						105	0	1350	1350	150	0
**1401**						87	-	0	0	1350	150
**1401**						102	-	1350	450	1350	50
**1432**	54	M	NO	1	NO	58	80	1350	1350	1350	150
**1432**						84	40	1350	450	450	50
**1457**	23	M	NO	1	NO	80	-	1350	1350	150	0
**1457**						87	-	450	150	150	0
**1457**						94	-	1350	1350	450	50
**1457**						101	-	450	1350	50	0
**1457**						108	-	1350	450	150	0
**1457**						122	-	1350	1350	150	0
**1462**	70	M	NO	1	NO	72	-	1350	1350	450	50
**1462**						79	80	1350	1350	450	50
**1462**						86	-	450	450	450	50
**1462**						93	-	1350	450	450	50
**1462**						100	20	150	1350	450	0
**1462**						107	-	1350	450	150	0
**1462**						114	0	450	1350	450	50
**1499**	42	M	NO	1	NO	79	20	1350	1350	450	50
**1499**						94	0	1350	450	450	0
**1551**	50	M	YES	5	YES	65	-	1350	1350	4050	450
**1551**						70	320	1350	1350	4050	450
**1551**						76	-	1350	1350	1350	150
**1551**						83	-	1350	1350	4050	450
**1551**						89	320	1350	1350	4050	450
**1551**						97	-	1350	1350	0	0
**1551**						104	-	1350	1350	1350	150
**1551**						111	160	1350	1350	450	150
**1678**	48	F	NO	2	YES	54	-	1350	1350	450	150
**1678**						85	-	1350	1350	1350	450
**1678**						92	-	1350	1350	1350	150
**1817**	28	F	NO	1	NO	44	-	1350	1350	450	150
**1817**						51	-	1350	1350	1350	450
**1817**						63	-	1350	1350	1350	450

DPO Days post onset of symptoms; S/ECD Spike ectodomain; S/RBD Spike receptor-binding domain; VN Virus neutralization
