# Supplementary material for: Limited window for donation of convalescent plasma with high live-virus neutralizing antibody titers for COVID-19 immunotherapy
Source: Commun Biol. 2021 Feb 24;4:267. doi: 10.1038/s42003-021-01813-y (PMC7904946; doi:10.1038/s42003-021-01813-y)
Supplement: Supplementary file 3 — Supplementary Data 1 [file 42003_2021_1813_MOESM3_ESM.docx]

**Supplementary Data 1:** **Predictive values and likelihood ratios of the ELISA methods as a surrogate for virus neutralizing antibody titer of ≥160.** Positive and negative predictive values, and Likelihood ratios (95% CI) has been provided for the various DPO categories.

| DPO | Effect | S/ECD ≥1350 | | S/RBD ≥1350 | | S/RBD IgG ≥1350 | | S/RBD IgM ≥450 | |
| --- | --- | --- | --- | --- | --- | --- | --- | --- | --- |
|  |  | **Value** | **95% CI** | **Value** | **95% CI** | **Value** | **95% CI** | **Value** | **95% CI** |
| Overall  0-142 | PPV | 0.52 | 0.46 to 0.59 | 0.57 | 0.50 to 0.63 | 0.72 | 0.65 to 0.79 | 0.38 | 0.30 to 0.46 |
|  | NPV | 0.75 | 0.65 to 0.83 | 0.87 | 0.78 to 0.92 | 0.82 | 0.75 to 0.87 | 0.90 | 0.84 to 0.94 |
|  | LR+ | 1.34 |  | 1.61 |  | 3.18 |  | 3.72 |  |
|  | LR- | 0.40 |  | 0.19 |  | 0.26 |  | 0.69 |  |
| 1-30 | PPV | 0.92 | 0.65 to 1.00 | 0.94 | 0.72 to 0.99 | 0.78 | 0.55 to 0.91 | 1.00 | 0.68 to 1.00 |
|  | NPV | 0.67 | 0.47 to 0.82 | 0.80 | 0.58 to 0.92 | 0.65 | 0.43 to 0.82 | 0.56 | 0.39 to 0.73 |
|  | LR+ | 9.85 |  | 13.43 |  | 2.83 |  | - |  |
|  | LR- | 0.45 |  | 0.22 |  | 0.44 |  | 0.62 |  |
| 31-60 | PPV | 0.79 | 0.67 to 0.88 | 0.69 | 0.57 to 0.78 | 0.73 | 0.61 to 0.82 | 0.90 | 0.74 to 0.96 |
|  | NPV | 0.56 | 0.41 to 0.70 | 0.72 | 0.52 to 0.83 | 0.72 | 0.55 to 0.84 | 0.55 | 0.43 to 0.66 |
|  | LR+ | 1.80 |  | 1.56 |  | 1.92 |  | 6.23 |  |
|  | LR- | 0.38 |  | 0.28 |  | 0.28 |  | 0.59 |  |
| 61-90 | PPV | 0.32 | 0.23 to 0.43 | 0.35 | 0.25 to 0.47 | 0.58 | 0.41 to 0.72 | 0.50 | 0.31 to 0.69 |
|  | NPV | 1.00 | 0.65 to 1.00 | 1.00 | 0.74 to 1.00 | 0.90 | 0.79 to 0.96 | 0.78 | 0.66 to 0.86 |
|  | LR+ | 1.13 |  | 1.30 |  | 3.20 |  | 2.40 |  |
|  | LR- | 0.00 |  | 0.00 |  | 0.27 |  | 0.69 |  |
| 91-120 | PPV | 0.52 | 0.40 to 0.64 | 0.61 | 0.48 to 0.73 | 0.87 | 0.71 to 0.95 | 0.80 | 0.49 to 0.96 |
|  | NPV | 1.00 | 0.77 to 1.00 | 1.00 | 0.85 to 1.00 | 0.87 | 0.74 to 0.94 | 0.62 | 0.50 to 0.73 |
|  | LR+ | 1.43 |  | 2.05 |  | 8.80 |  | 5.21 |  |
|  | LR- | 0.00 |  | 0.00 |  | 0.20 |  | 0.79 |  |
| >120 | PPV | 0.43 | 0.16 to 0.75 | 0.43 | 0.16 to 0.75 | 0.75 | 0.30 to 0.99 | 0.00 | 0.00 to 0.95 |
|  | NPV | 1.00 | 0.05 to 1.00 | 1.00 | 0.05 to 1.00 | 1.00 | 0.51 to 1.00 | 0.57 | 0.25 to 0.84 |
|  | LR+ | 1.25 |  | 1.25 |  | 5.00 |  | 0.00 |  |
|  | LR- | 0.00 |  | 0.00 |  | 0.00 |  | 1.25 |  |

DPO Days post onset of symptoms; S/ECD Spike ectodomain; S/RBD Spike receptor-binding domain; PPV Positive predictive value; NPV Negative predictive value; LR+ Positive likelihood ratio; LR- Negative likelihood ratio
