# Supplementary material for: Limited window for donation of convalescent plasma with high live-virus neutralizing antibody titers for COVID-19 immunotherapy
Source: Commun Biol. 2021 Feb 24;4:267. doi: 10.1038/s42003-021-01813-y (PMC7904946; doi:10.1038/s42003-021-01813-y)
Supplement: Supplementary file 4 — Reporting Summary [file 42003_2021_1813_MOESM4_ESM.pdf]

nature research

Corresponding author(s): Suresh Varma Kuchipudi, James Musser, Vivek Kapur

Last updated by author(s): Jan 12, 2021

Reporting Summary

Nature Research wishes to improve the reproducibility of the work that we publish. This form provides structure for consistency and transparency in reporting. For further information on Nature Research policies, see our Editorial Policies and the Editorial Policy Checklist.

Statistics

For all statistical analyses, confirm that the following items are present in the figure legend, table legend, main text, or Methods section.

n/a	Confirmed	
☐	☒	The exact sample size (*n*) for each experimental group/condition, given as a discrete number and unit of measurement
☐	☒	A statement on whether measurements were taken from distinct samples or whether the same sample was measured repeatedly
☐	☒	The statistical test(s) used AND whether they are one- or two-sided<br>*Only common tests should be described solely by name; describe more complex techniques in the Methods section.*
☒	☐	A description of all covariates tested
☒	☐	A description of any assumptions or corrections, such as tests of normality and adjustment for multiple comparisons
☐	☒	A full description of the statistical parameters including central tendency (e.g. means) or other basic estimates (e.g. regression coefficient) AND variation (e.g. standard deviation) or associated estimates of uncertainty (e.g. confidence intervals)
☐	☒	For null hypothesis testing, the test statistic (e.g. *F*, *t*, *r*) with confidence intervals, effect sizes, degrees of freedom and *P* value noted<br>*Give P values as exact values whenever suitable.*
☒	☐	For Bayesian analysis, information on the choice of priors and Markov chain Monte Carlo settings
☒	☐	For hierarchical and complex designs, identification of the appropriate level for tests and full reporting of outcomes
☒	☐	Estimates of effect sizes (e.g. Cohen's *d*, Pearson's *r*), indicating how they were calculated

Our web collection on statistics for biologists contains articles on many of the points above.

Software and code

Policy information about availability of computer code

Data collection	No software was used in the data collection
Data analysis	Statistical analysis of the results were performed with Graphpad PRISM v 8.4.3 and R software version 1.2.5019

For manuscripts utilizing custom algorithms or software that are central to the research but not yet described in published literature, software must be made available to editors and reviewers. We strongly encourage code deposition in a community repository (e.g. GitHub). See the Nature Research guidelines for submitting code & software for further information.

Data

Policy information about availability of data

All manuscripts must include a data availability statement. This statement should provide the following information, where applicable:

- Accession codes, unique identifiers, or web links for publicly available datasets
- A list of figures that have associated raw data
- A description of any restrictions on data availability

All the raw data used in the study is provided as supplementary tables and figures

Field-specific reporting

Please select the one below that is the best fit for your research. If you are not sure, read the appropriate sections before making your selection.

☒ Life sciences ☐ Behavioural & social sciences ☐ Ecological, evolutionary & environmental sciences

For a reference copy of the document with all sections, see nature.com/documents/nr-reporting-summary-flat.pdf

Life sciences study design

All studies must disclose on these points even when the disclosure is negative.

Sample size	The study cohort consisted of 540 plasma samples collected from 175 SARS-CoV-2 convalescent patients. Of the 175 subjects, 105 individuals donated convalescent plasma at least twice (range 2-12 times). All the donors have been confirmed negative for SARS-CoV-2 by RT-PCR before plasmapheresis. There was no statistical analysis performed to determine the sample size.
Data exclusions	Total antibody ELISA to SARS-CoV-2 spike-ectodomain (S/ECD) and spike-receptor binding domain (S/RBD) was performed on 538 samples, and specific isotype antibody IgG and IgM ELISA were performed on 540 samples. A sub-set of 305 samples were analyzed by virus neutralization assays.
Replication	The reproducibility of the ELISA methods were tested in duplicate. The results from the ELISA methods were tested to have a strong correlation among the assays. The antibody titers were tested once per assay and declared as ODs or titers.
Randomization	No randomization is required in the study design.
Blinding	The samples were blinded to personnel who performed the assays (ELISAs and virus neutralization). The results were then matched to the IDs and then declared.

Reporting for specific materials, systems and methods

We require information from authors about some types of materials, experimental systems and methods used in many studies. Here, indicate whether each material, system or method listed is relevant to your study. If you are not sure if a list item applies to your research, read the appropriate section before selecting a response.

Materials & experimental systems

n/a	Involved in the study	
☐	☒	Antibodies
☐	☒	Eukaryotic cell lines
☒	☐	Palaeontology and archaeology
☒	☐	Animals and other organisms
☐	☒	Human research participants
☒	☐	Clinical data
☒	☐	Dual use research of concern

Methods

n/a	Involved in the study	
☒	☐	ChIP-seq
☒	☐	Flow cytometry
☒	☐	MRI-based neuroimaging

Antibodies

Antibodies used	CR3022 IgG1: Ab01680-10.0 & CR3022 IgM: Ab01680-15.0, Absolute antibody, USA; anti-human IgG Fab HRP conjugate (A0293, Sigma Aldrich); Peroxidase conjugated anti-Human IgM (Fc5µ) antibody produced in rabbit (SAB3701404, Sigma Aldrich); Peroxidase conjugated anti-human IgG (Fc specific) antibody produced in goat (A0170, Sigma Aldrich)
Validation	The antibodies used were verified and validated by the manufacturers.

Eukaryotic cell lines

Policy information about cell lines

Cell line source(s)	Vero E6 cells were obtained from ATCC
Authentication	Authenticated by ATCC
Mycoplasma contamination	Declared negative by ATCC
Commonly misidentified lines (See ICLAC register)	None

Human research participants

Policy information about studies involving human research participants

Population characteristics	Plasma samples (n=540) from 175 COVID-19 convalescent patients collected at Houston Methodist Hospital in Houston, Texas were studied. The study cohort consisted of 88 females (50.3%) and 87 males (49.7%), ranging in age between 20-78 years (median 46, IQR: 36-54). Samples were collected from 17-142 DPO (median 68 days, IQR: 48-93).
Recruitment	All the patients were tested positive for SARS-CoV-2 infection by RT-PCR at Houston Methodist hospital. As per FDA guidelines, all subjects were asymptomatic for at least 14 days at the time of plasma sample collection. Informed consent was obtained from either the patient or an authorized representative of the patient, when applicable for the collection of plasma samples.
Ethics oversight	Houston Methodist hospital, Texas (IRB# PRO00025121), Pennsylvania State University's Institutional Biosafety Committee

Note that full information on the approval of the study protocol must also be provided in the manuscript.
